# Can Combining Hyaluronic Acid and Physiotherapy in Knee Osteoarthritis Improve the Physicochemical Properties of Synovial Fluid?

**DOI:** 10.3390/biomedicines12020449

**Published:** 2024-02-17

**Authors:** Ilie Onu, Robert Gherghel, Isabella Nacu, Florina-Daniela Cojocaru, Liliana Verestiuc, Daniela-Viorelia Matei, Dan Cascaval, Ionela Lacramioara Serban, Daniel Andrei Iordan, Alexandra Tucaliuc, Anca-Irina Galaction

**Affiliations:** 1 Department of Biomedical Sciences, Faculty of Medical Bioengineering, University of Medicine and Pharmacy “Grigore T. Popa”, 700454 Iasi, Romania; ilie.onu@umfiasi.ro (I.O.); cobzariu.isabella@gmail.com (I.N.); florina.cojocaru@umfiasi.ro (F.-D.C.); liliana.verestiuc@bioinginerie.ro (L.V.); daniela.matei@umfiasi.ro (D.-V.M.); anca.galaction@umfiasi.ro (A.-I.G.); 2 Department of Physiotherapy, Micromedica Clinic, 610119 Piatra Neamt, Romania; 3 Department of Morpho-Functional Sciences II, Faculty of Medicine, University of Medicine and Pharmacy “Grigore T. Popa”, 700115 Iasi, Romania; ionela.serban@umfiasi.ro; 4 Petru Poni Institute of Macromolecular Chemistry, 41-A Grigore Ghica Voda Alley, 700487 Iasi, Romania; 5 Department of Organic, Biochemical and Food Engineering, Faculty of Chemical Engineering and Environmental Protection “Cristofor Simionescu”, Technical University “Gheorghe Asachi”, 700050 Iasi, Romania; dan.cascaval@academic.tuiasi.ro (D.C.); alexandra.tucaliuc@academic.tuiasi.ro (A.T.); 6 Department of Individual Sports and Kinetotherapy, Faculty of Physical Education and Sport, “Dunarea de Jos” University of Galati, 800008 Galati, Romania; 7 Center of Physical Therapy and Rehabilitation, “Dunărea de Jos” University of Galati, 800008 Galati, Romania

**Keywords:** hyaluronic acid, synovial fluid, ATR-FTIR spectroscopy, rheological properties, bioadhesion, physical therapy, knee osteoarthritis

## Abstract

Known as the degenerative disease of the knee with the highest prevalence, knee osteoarthritis (KOA) is characterized by a gradual destructive mechanism that, in severe cases, can provoke the need for total knee substitution. As the disease progresses, various enzymatic, immunological, and inflammatory processes abnormally degrade hyaluronic acid (HA), SF’s main component, and affect the concentrations of specific proteins, with the final results seriously endangering synovial fluid (SF)’s rheological and tribological features and characteristics. No effective treatments have been found to stop the progression of KOA, but the injection of HA-based viscoelastic gels has been considered (alone or combined with physiotherapy (PT)) as an alternative to symptomatic therapies. In order to evaluate the effect of viscosupplementation and PT on the characteristics of SF, SF aspirated from groups treated for KOA (HA Kombihylan^®^ and groups that received Kombihylan^®^ and complex PT) was analyzed and compared from analytical, spectrophotometrical, and rheological perspectives. In the patients treated with PT, the SF extracted 6 weeks after viscosupplementation had a superior elastic modulus (G′) and viscous moduli (G″), as well as a homogeneous distribution of proteins and polysaccharides. The viscosupplementation fluid improved the bioadhesive properties of the SF, and the use of the viscosupplementation fluid in conjunction with PT was found to be favorable for the distribution of macromolecules and phospholipids, contributing to the lubrication process and the treatment of OA-affected joints.

## 1. Introduction

Arthritis is a major health problem. It has a prevalence higher than cancer, diabetes, and AIDS, and with the global phenomenon of population aging, its incidence is rising [1]. It is recognized as a chronic disabling disease with several clinical symptoms, including pain, stiffness, swelling, deformity, and necrosis [2]. More than 100 forms of arthritis have been identified, but most forms, including osteoarthritis (OA) and rheumatoid arthritis (RA), synovitis and gout, bursitis, and traumatic arthritis, include joint inflammation [3]. This alarming situation necessitates intensive studies for prevention, precise and early diagnoses, and more efficient treatment for patients [4]. 

**Figure 1:**
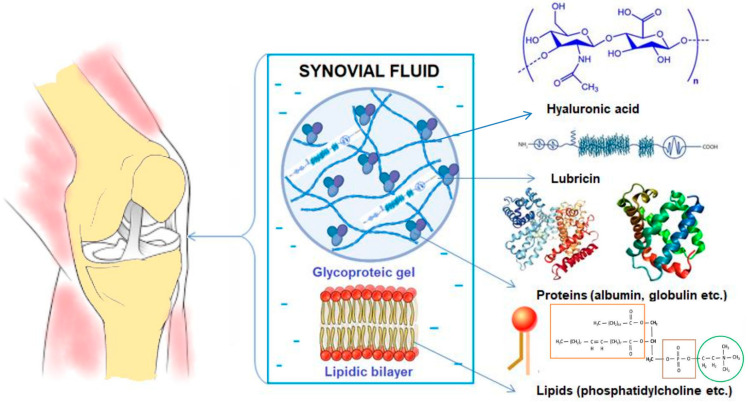
Synovial fluid (SF) components.

OA is an all-articular disease that generally affects elderly patients, and it involves articular cartilage, subchondral bone, ligaments, capsules, synovial joints, and periarticular muscles; generally, anatomical structures are destroyed and lose their function, ultimately leading to joint failure. OA causes various changes in the joint over time, manifested by the erosion of the articular cartilage, meniscal lesions, synovitis, subchondral bone sclerosis, and spurs that will irritate the tendons, limiting mobility and generating pain. Joint pain is associated with the alteration of synovial fluid (SF), leading to failures in the lubrication of the boundaries that prevent direct contact between bones [5,6,7]. Therefore, the primary purposes of SF are to serve as a synovial lubricant to help synovial joints absorb shock and to offer a source of nutrition for the joint cartilage (Figure 1) [8].

One key component of SF is hyaluronic acid (HA), a high-molecular-weight polysaccharide involved in a wide range of physiological processes in the human body, including wound healing, tumor progression, and joint lubrication. HA interacts with lipid membranes and contributes to joint lubrication [9,10,11]. Degenerated SF experiences changes in the concentrations of its constituents and alterations in its rheological properties, including reduced viscosity compared to healthy SF, and all of these effects play a decisive role in the development of joint diseases [12].

Since there is a lack of knowledge regarding the mechanism of OA [13], different approaches have been used to address this much-discussed pathology, but most have yielded only modest results. Therapies and medications aiming to restore joint structures or at least to preserve the integrity of joint structures, relieve pain and inflammation, and reduce dysfunction have been tested, with few side effects being observed alongside long-term costs [4].

Regarding medication, simple analgesics and non-steroidal anti-inflammatory drugs (NSAIDs), used sometimes in combination with a selective competitive inhibitor of cyclooxygenase, COX-2, are frequently used [14]. Even persistent pain, which results in a poor quality of life, can be relieved by high doses of NSAIDs, but their long-term use can bring serious side effects, such as gastric ulcers, renal failure, or even stroke [14,15]. To avoid the side effects of these orally administrated drugs, some clinicians resort to the use of more invasive therapies that yield targeted results, such as viscosupplementation [16]. This technique involves administering intra-articular injections of HA, with the aim being to reinforce the SF’s physiologic viscoelasticity. At the molecular level, HA has a great ability to diminish the expression levels of pro-inflammatory factors (prostaglandin E2–PGE2 and nuclear factor kappa-light-chain-enhancer of activated B cells–NF-κB) and enzymes known to non-specifically degrade joint matrices in pathological conditions. Like any other injection therapy, HA supplementation has some contraindications and side effects, but in comparison with therapeutic strategies involving NSAIDs, these effects are local, mild, and transient. Moreover, HA supplementation is more efficient than the use of NSAIDs in terms of pain diminution and enhancing functionality [17]. Regarding HA viscosupplementation in knee osteoarthritis (KOA) management, there is no consensus between national and international guidelines on whether the use of this method is recommended. For example, some guidelines recommend it after the use of NSAIDs, while others are against it. The American College of Rheumatology recommends the use of viscosupplementation for patients with contraindications for surgery or for those who have a negative response to other approaches. Some European expert groups frame HA intra-articular injection as a preventive method based on its chondroprotective effects [18].

Leaving aside the pharmacological therapies mentioned before, primordial to symptom management are patient education, weight loss, and, perhaps most important, physical activity. Practicing mild-intensity aerobic exercises for 150 min weekly or, if possible, mild to strong muscle-strengthening exercises contributes to preventing KOA [19]. 

Non-invasive non-pharmacological therapies (NINPTs) are seen as adequately safe solutions for chronic pain, preserving the tissue without significant side effects. They can be divided into the following categories: physical therapies (PTs), psychological interventions, and alternative and complementary approaches. PTs include transcutaneous electrical nerve stimulation (TENS), heat therapy and cryotherapy, massages [20], ultrasound (US), and low-level laser therapy (LLLT) [21]. The present psychological interventions are based on positive and cognitive behavioral changes, while the current alternative and complementary approaches are a mixture of PT and psychological interventions (e.g., yoga, acupuncture, etc.). 

An effective alternative with limited side effects in the management of KOA is the administration of HA-based viscoelastic substances via intra-articular injection and physical therapy (PT). Although many types of PT have been carefully studied, currently, there are no guidelines for standardized PT protocols using effective combinations of physical agents and medications to control the progression of KOA [16]. Both HA viscosupplementation and PT have anti-inflammatory effects on low-grade inflammation in KOA, and combining the two therapies can prolong the effects of HA viscosupplementation [19]. Understanding the biochemical and biophysical mechanism of reducing pain using NINPTs, with or without pharmacological therapies, is essential in enhancing their clinical application [20].

TENSs are rectangular single-phase or biphasic pulsed currents that are distributed across the skin to stimulate the underlying nerves and produce intense analgesia [22]. A TENS machine selectively activates the non-nociceptive Aβ-fibers, which are large-diameter fibers that have the fastest conduction velocity of all fibers in the body. TENS treatment has been found to diminish nociceptor sensitivity and activity at a segmental level and primary sensory neurons, reducing pain in KOA and reducing inflammation by lowering pro-inflammatory cytokine (IL) levels (especially IL-6) through two possible mechanisms: (1) activating the release of opioids by the central nervous system and (2) the pain gate mechanism (gate theory) [23,24,25,26,27].

US is a high-frequency therapy that uses mechanical vibrations with frequencies between 1 and 3 MHz to stimulate the cellular and molecular mechanisms involved in healing processes [28]; in the context of KOA, it can control symptoms and potentially play a role in cartilage repair [29,30,31,32]. 

LLLT, not included yet in current KOA treatment protocols, is a form of low-power laser photo biostimulation that exerts significant analgesic and anti-inflammatory actions and a biomodulatory effect on the microcirculation, which helps heal tissues and reduce lymphoedema [33,34,35]. In a systematic review and meta-analysis of placebo-controlled randomized clinical trials (RCTs) published in 2019, Stausholm MB et al. highlighted that LLLT significantly reduces pain and inflammation in KOA cases [36].

Cryotherapy, also not usually found in KOA treatment guidelines [37], is a safe and widely used PT procedure in the control of post-traumatic pain, inflammation, and edema management. It can be used to lower the expression levels of pro-inflammatory cytokines (mainly tumor necrosis factor α–TNF-α and interleukin 10–IL-10) [38,39,40,41]. Cryotherapy was used in this study to reduce joint temperature, especially after exercise, and to protect intra-articular-injected HA biopolymers. 

Guidelines from the American Academy of Orthopedic Surgeons, the American College of Rheumatology, and the European League against Rheumatism are consistent in recommending physical exercise (PE) because physical activity can relieve pain, improve mobility, and increase one’s quality of life [42,43,44,45]. Isokinetic PE is an effective form of training for the toning of the quadricep muscles that is useful in the management of KOA, exerting an anti-inflammatory effect by reducing the serum levels of C-reactive protein, TNF-α, and IL-6 [46]. 

All the NINPTs presented here as part of KOA treatment strategies have certain barriers and drawbacks, and overall, the treatment landscape bears disadvantages related to heterogeneity and individualization, the lack of treatment standardization, affordability, and adherence, and research methodology restrictions in studies to improve on the current treatments [20]. 

Considering all theoretical aspects mentioned already, the objective of this study was to evaluate the ability of a combination therapy, namely the combination of intra-articular HA supplementation (Kombihylan^®^) and PT, to improve the properties of SF from knees with moderate KOA. The PT program included TENS currents, laser photo-stimulation, low-dose ultrasound, exercises, and cryotherapy. We set out guided by the notion that understanding the biochemical and biophysical mechanism of reducing pain using NINPTs with or without pharmacological therapies is essential to enhance their clinical application [20]. In the existing literature, studies on NINPTs are limited, with some of the few studies that have been conducted on this topic presenting incomplete data, which supports and highlights the novelty and importance of this study.

## 2. Materials and Methods

### 2.1. Materials

Commercially available Kombihylan^®^ viscoelastic material was purchased from Rompharm Company^®^ (Romania) and used after approval for its use was granted (with approval no. 11306 (2020)). Kombihylan^®^ is a biological matrix with a molecular weight of 3 MDa, and it comes in the form of a viscoelastic solution containing crosslinked HA (sodium salt, 16 mg/mL) obtained via the bacterial fermentation of a Streptococcus strain, chondroitin sodium sulfate (30 mg/mL), N-acetyl D-glucose amine (30 mg/mL), sodium salts (NaCl–4.3 mg/mL; NaH_2_PO_4_ × 2H_2_O–0.52 mg/mL; Na_2_HPO_4_ × 12H_2_O–5.04 mg/mL), and NaOH/H_3_PO_4_ (up to pH 7.4). Our study was designed according to the guidelines of the Declaration of Helsinki and approved by the Institutional Review Board (or Ethics Committee) of Micromedica Medical Clinic (approval no. 32) on 28 January 2020. All patients signed an informed consent form. The product was administered intra-articularly to patients with moderate KOA by an orthopedic specialist.

### 2.2. Study Design and Procedure Steps (PSs)

SF samples were collected from nine patients who had been divided into 3 groups. In the control group, no procedure (*n* = 3) was applied, and in the other two groups, 2–3 mL of SF was aspirated (Control 1, Control 2, and Control 3). 

**Figure 2:**
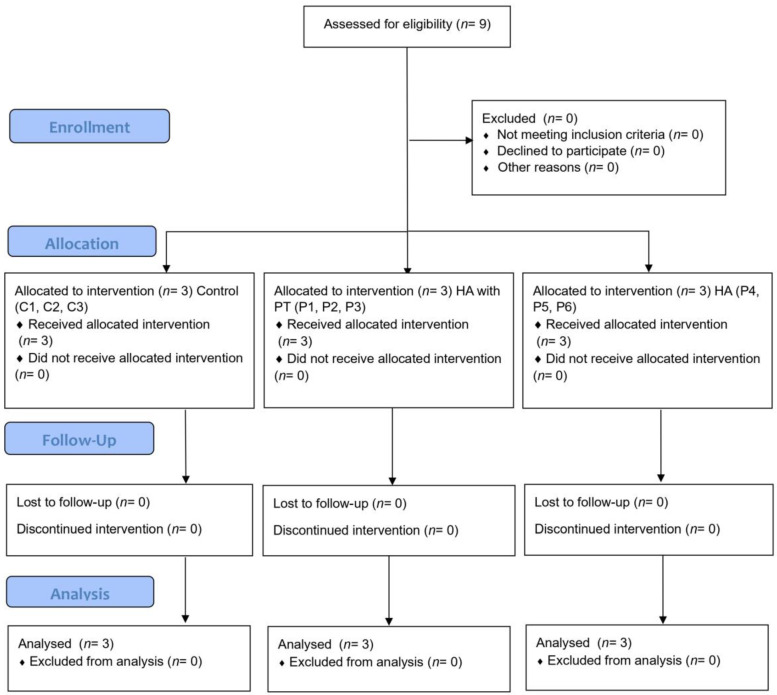
Flowchart of the study design and the groups of patients.

Regarding our inclusion criteria, patients were considered eligible if they had been diagnosed with symptomatic moderate KOA with no signs of local inflammation and infiltrations with other viscoelastic substances or glucocorticoids in the past 12 months. Patients with only one symptomatic knee were also included. Our exclusion criteria were as follows: known allergy or hypersensitivity to sodium hyaluronate (or any of the ingredients in Kombihylan^®^); patients with signs of local inflammation or hydrarthrosis, infections at the injection site, or skin diseases at the injection site; patients with inflammatory rheumatic disease or systemic disease; and patients with known systemic bleeding disorders. The study design and the groups of patients are presented in the flowchart below (Figure 2).

**Figure 3:**
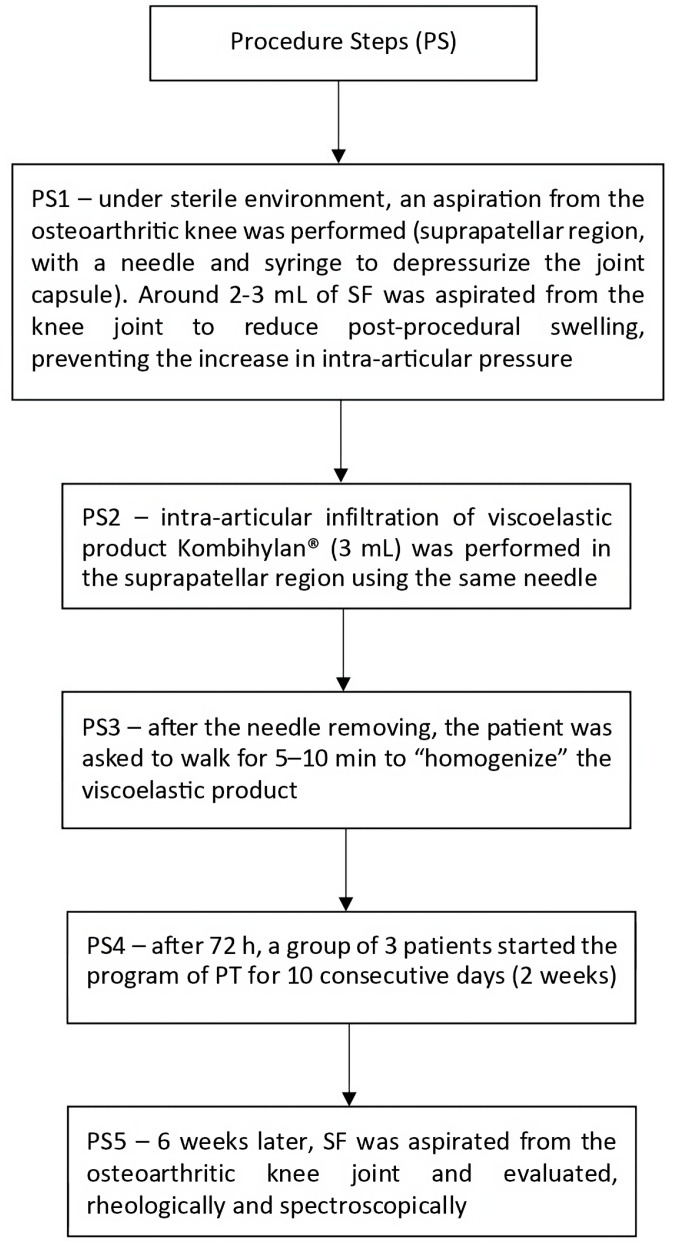
Procedural steps followed to carry out viscosupplementation.

Regarding viscosupplementation, five steps (PS1–PS5) were followed in order to perform experiments; these steps are summarized in Figure 3.

### 2.3. Physiotherapy Treatment

Physiotherapy treatment included the following procedures:

- PT1—conventional TENS for 30–40 min, two channels, 100 Hz, 100 µs rectangular biphasic pulses IONOSON-Expert (PHYSIOMED ELEKTROMEDIZIN AG Schnaittach, Germany);
- PT2—LLLT, 904 nm GaAlAs probe, 3 kHz frequency with 5 J/point and a maximum of 40 J/application (Pagani Roland LASER IR27, Fimad Elettromedicali, Catanzaro, Italy);
- PT3—8 min of US, 0.2–0.3 W/cm^2^ at 1 MHz with a 10% duty cycle IONOSON-Expert (PHYSIOMED ELEKTROMEDIZIN AG Schnaittach, Germany);
- PT4—over 40 min (per session) of moderate-intensity PE that included active exercises, isokinetic exercises, isometry, and neuro-proprioceptive facilitation (PNF, contract–relax, reversal of antagonists, repeated stretching, and hold–relax stretching);
- PT5—15 min CryoPush therapy (CRYOPUSH Cold Compression Therapy System, Sichuan, PRC).

### 2.4. Synovial Fluid’s Characteristics, pH, and Glucose Content

Immediately after aspiration, the SF samples were macroscopically evaluated in terms of color and clarity, with these details providing early indications of the disease. An increased level of turbidity suggested inflammation or infection [47]. 

SF pH measurements were carried out using a multi-parameter pH meter (HI2020–edge*^®^* Multiparameter pH Meter, Hanna Instruments, Woonsocket, RI, USA). A three-point calibration (pH = 4.01, 7.00, and 10.01) using standard buffer solutions (Hanna Instruments, Woonsocket, RI, USA) was performed. Measurements were performed at room temperature after macroscopic examination.

The concentration of glucose in the SF samples was measured by using a portable glucometer (Accu-chek Performa*^®^*, Roche Laboratories, Indianapolis, IN, USA) according to the manufacturer’s instructions. In brief, 1 µL SF was added on the front edge of the test strip, with the value (mg/dL) being shown on the device display after a certain time.

Before storing the fluid at 4 °C for further evaluation, 1 mL from each patient was added to the well of a sterile cell culture plate (24 wells) and incubated at 37 °C (48 h) in a drying oven (Electronic April, Cluj-Napoca, Romania). Finally, the dried solid was weighed using a 4-digit analytical balance (Kern, Balingen, Germany), with the results being displayed in mg/mL.

### 2.5. Drop Deposition of Synovial Fluid and Attenuated Total Reflectance Fourier-Transform Infrared Spectroscopy (ATR-FTIR)

The SF specimens were examined for their characteristics, and some preparative steps were performed. Small-volume drops (15 μL) of SF were deposited into 24-well plates for cell culturing at 37 °C, semi-covered, and allowed to dry overnight prior to being examined using light microscopy (Inverted-Phase Contrast Microscope, Leica, Wetzlar, Germany), stereomicroscopy (Stereomicroscope, Optika, Ponternica, Italy), and infrared spectroscopy. Attenuated total reflectance Fourier-transform infrared spectroscopy (ATR-FTIR; Nicolet Summit Pro FTIR Spectrometer with Everest ATR accessory, Thermo ScientificTM, Waltham, MA, USA) was used to evaluate the compositional changes in the synovial fluid. All samples were acquired using a diamond crystal at room temperature. Measurements were performed in the range of 400–4000 cm^−1^, with a spectral resolution of 4 cm^−1^ and 16 repetitious scans averaged for each spectrum. Prior to measurement, the materials were conditioned at 25 °C and 65% relative humidity for 24 h.

### 2.6. Rheological Measurements of Synovial Fluid

In this study, rheometry measurements were carried out using the Kinexus Pro+ rotational rheometer (Malvern Instruments Ltd., Worcestershire, UK), fitted with a parallel-plate geometry. All experiments were performed at a controlled temperature of 37 °C (with an accuracy of ± 0.1 °C using the Peltier system of the rheometers on the testing plate), and the experimental data were registered using rSpace for Kinexus Pro 1.7 software (rSpace Software, NETZSCH-Gerätebau GmbH, Germany). The amount of sample required for a rheological evaluation was 0.6 mL, and the remainder of each sample was used for our spectroscopic analysis and bioadhesion tests. To measure SF’s viscosity and viscoelastic properties, the steady shear test and oscillatory shear test were performed. The rheometer we used was suitable for determining the linear viscoelastic range (LVR) via a strain test performed using a 10 rad/s angular frequency (maintained constant) and a deformation amplitude ranging from 0.01 to 100%, while 1% amplitude (within LVR) and 0.01–200 rad/s frequency were the parameters for the frequency sweep tests. 

### 2.7. Bioadhesive Characteristics

Our bioadhesion tests were performed using a TA.XT Plus^®^ Texture Analyzer (Stable Micro Systems, Godalming, UK) fitted with a module for bioadhesion tests [48,49] on two types of surfaces: simulating biological membrane and chicken cartilage. A cellulose membrane from a 12,000 Da dialysis tube (pre-boiled and cooled at room temperature) was prepared for our in vitro experiments. Fresh cartilage was obtained from chicken femur condyle and specially prepared for our experiments. The cartilage samples were collected at least one week before the tests and kept hydrated in physiological solution, together with the synovial membrane, without freezing. 

Our tests were performed under physiological conditions. For each measurement, in the holding device, after adjusting one of the two surfaces, an adequate volume of a simulated body fluid with pH 7.2 and a concentration of 0.01 M (phosphate-buffered solution–PBS) was added as an immersion medium. During the test, the holding system was maintained in a 37 °C distilled water bath under 200 rpm stirring. The bioadhesion assay involved keeping the dried synovial fluid films (attached to an 8 mm Ø cylindrical rod) in contact with the cellulose membrane/chicken cartilage for 30 s (contact force: 9.80665 mN). The device-specific software (Texture exponent) provided the maximum detachment force and the work of adhesion. The results are presented as the mean ± standard deviation of six different measurements (to avoid substantial method errors).

### 2.8. Statistical Analyses 

With the exception of the bioadhesion assays, all assays were performed in triplicate, and the results are displayed as the mean ± standard deviation. Where applicable, we conducted a one-way ANOVA and Tukey’s post hoc analysis for our statistical analyses (*p* < 0.05).

## 3. Results

### 3.1. Synovial Fluid Characteristics

Our laboratory analysis for all tested SF samples showed amber–yellow or yellow-colored and transparent fluid (Table 1), indicating OA as a joint disease.

**Figure 4:**
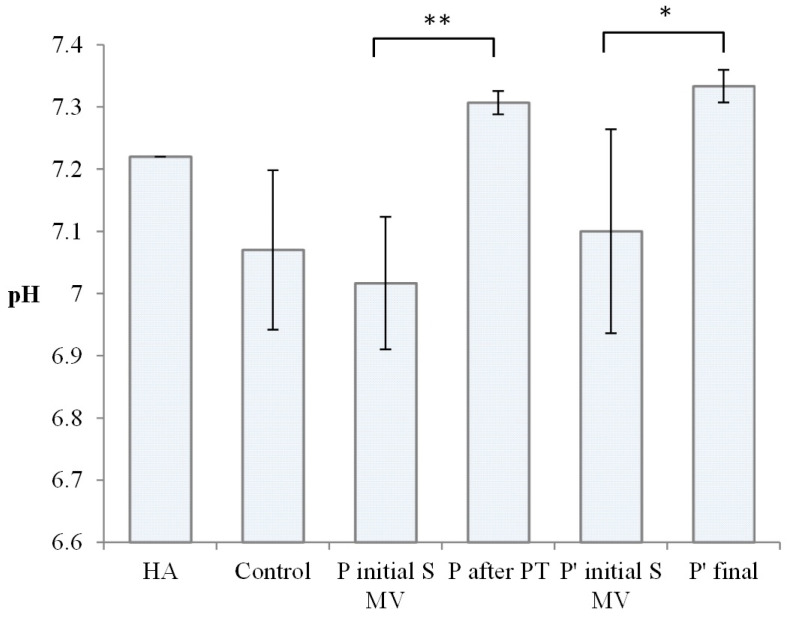
Value of pH for Kombihylan^®^ and synovial fluid (SF) from the control group, Group A (supplemented, 5–10 min movement), Group B (after PT; 2 weeks PT + 4 weeks rest), Group C (supplemented, 5–10 min movement), and Group D (supplemented, 5–10 min movement, 6 weeks rest). Each value represents the mean ± standard error of the mean (*n* = 3) (* *p* < 0.05, ** *p* < 0.01).

The pH of normal SF is 7.31–7.64, with the mean pH being 7.43. Under conditions of inflammation, on average, the pH declines to 7.22 or broadly ranges from 6.85 to 7.41 for joint diseases, as reported in [50]. The pH values of the tested SF samples are presented in Figure 4.

The SF samples in the control group presented a wide distribution of pH values, with a mean of 7.07, in agreement with other authors’ findings regarding the modification of SF’s pH in OA development [51]. pH values between 7 and 7.2 were also recorded for the SF samples from Group A (supplemented, 5–10 min movement) and Group C (supplemented, 5–10 min movement). In Group B (2 weeks PT + 4 weeks rest) and Group D (supplemented, 5–10 min movement, 6 weeks rest), after PT, the pH of the synovial fluid approached normal values; in terms of pH, both PT and long resting are effective, suggesting complex time-dependent influences. 

High variability in the glucose contents of the SF samples was observed, with the highest values corresponding to those in the control group. 

### 3.2. Fourier-Transform Infrared (FTIR) Data

**Figure 5:**
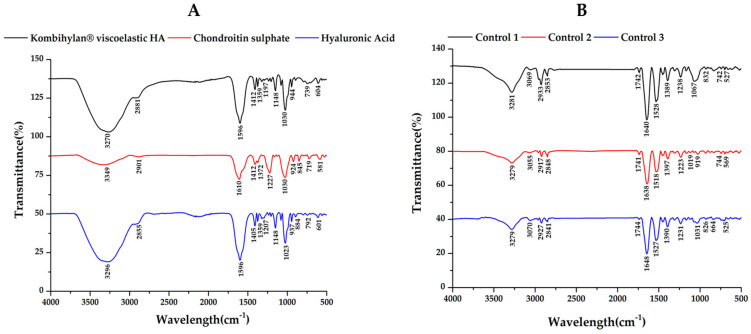
ATR−FTIR spectra for (**A**) hyaluronic acid (HA), chondroitin sulfate (CS), and the viscosupplementation product, as well as for the (**B**) synovial fluid (SF) samples collected from the control group.

In order to evaluate the effect of viscosupplementation and PT, the SFs aspirated from the osteoarthritic knee joint were spectrophotometrically analyzed via ATR-FTIR and compared according to the control group, viscosupplementation material, HA, and chondroitin sulfate. The first step was to analyze the spectra of the SF’s main polymers and those of the viscosupplementation product (Figure 5A and Table 2).

**Figure 6:**
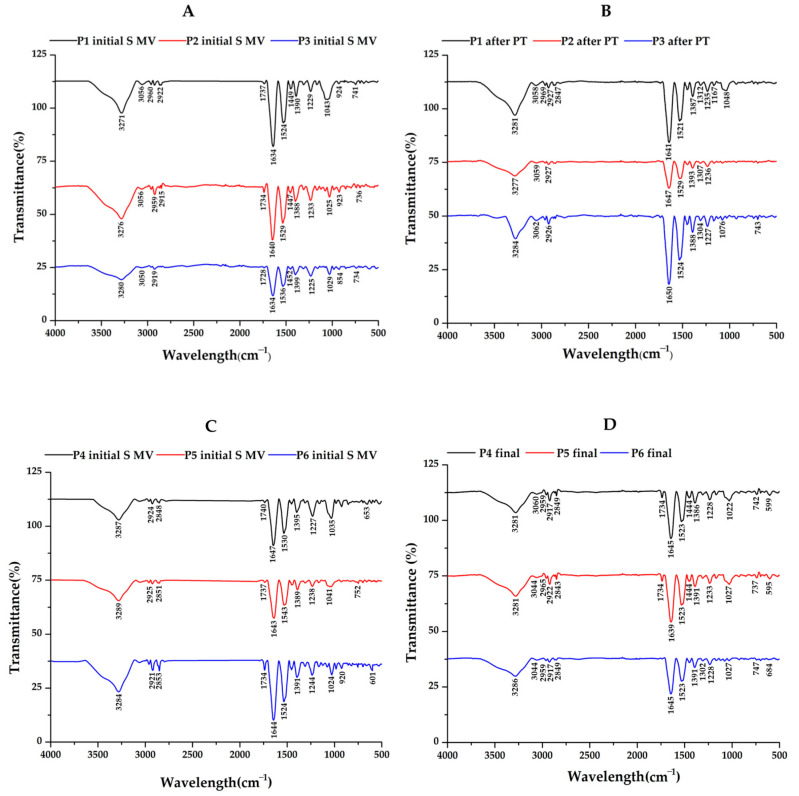
ATR−FTIR spectra for synovial fluid (SF) samples collected from patients assigned to (**A**) Group A (supplemented, 5–10 min movement); (**B**) Group B—after PT (2 weeks PT + 4 weeks rest); (**C**) Group C (supplemented, 5–10 min movement); and (**D**) Group D (supplemented, 5–10 min movement, 6 weeks rest).

Regarding the control group (Figure 5B), decreased absorption in the 980–1140 cm^−1^ domain corresponding to the carbohydrate content (sugar bands) was identified, along with an overexpression of the amide I region (1584–1720 cm^−1^), indicating an increased total protein content based on the composition of the SF. Also, the sugar bands in the 980–1140 cm^−1^ domain were less intense for the SF samples collected from the patients treated via supplementation, 5–10 min of movement, and PT treatment (PT, 2 weeks) before 4 weeks of resting compared to those who did not have PT treatment (Figure 6A,B). The intensity of the sugar absorption did not change for those in Groups C (supplemented and 5–10 min movement—Figure 6C) and D (supplemented and 5–10 min movement, 6 weeks rest—Figure 6D) [55]. 

### 3.3. Rheological Properties

**Figure 7:**
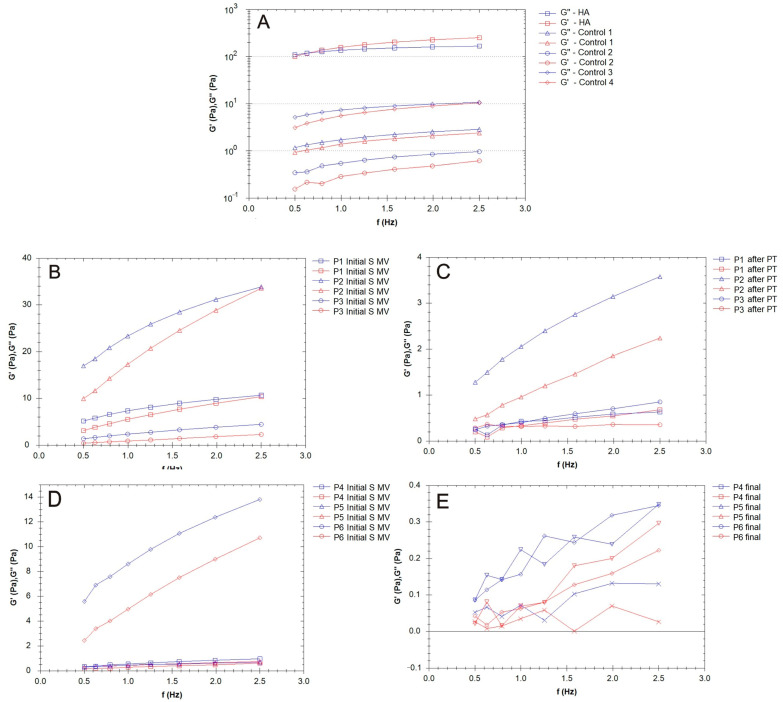
Elastic (G′) and viscous (G″) moduli for the synovial fluid (SF) samples collected from the control group (CG) and those who received the viscosupplementation product (Group A). (**A**) Patients treated supplementation, 5–10 min movement, and PT (2 weeks PT + 4 weeks rest); (**B**,**C**) patients treated via supplementation and 5–10 min movement; and (**D**,**E**) patients treated via supplementation, 5–10 min movement, and 6 weeks of rest.

**Figure 8:**
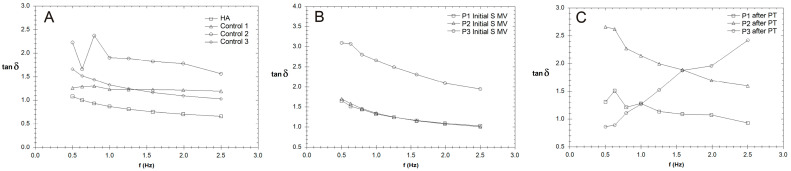
The variation in tan δ with frequency (Hz) for synovial fluid (SF) collected from the control group (CG) and those who received the viscosupplementation product (**A**), as well as the patients who were treated via supplementation, 5–10 min movement, and PT (2 weeks PT + 4 weeks rest) (**B**,**C**).

**Figure 9:**
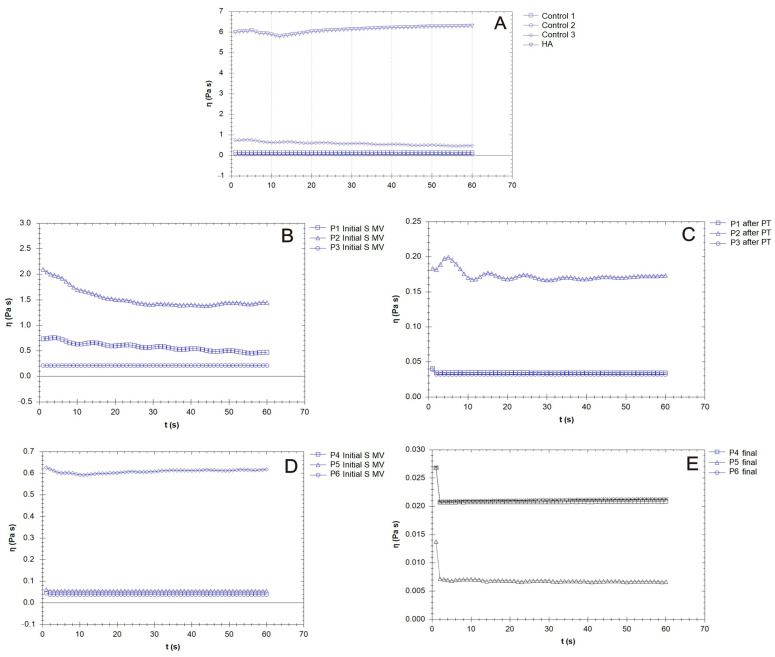
The variation in viscosity for the synovial fluid (SF) samples collected from the control group (CG) and those who received the viscosupplementation product (**A**); the patients treated via supplementation, 5–10 min movement, and PT (2 weeks PT + 4 weeks rest) (**B**,**C**); and the patients treated supplementation, 5–10 min movement and supplementation, 5–10 min movement, and 6 weeks rest (**D**,**E**).

In the present study, the rheological properties of SF samples extracted from patients with moderate KOA were investigated after the application of different treatments, including the following treatments: supplementation, 5–10 min movement, and PT (2 weeks PT + 4 weeks rest); supplementation and 5–10 min movement; and supplementation, 5–10 min movement, and 6 weeks of rest (Figure 7, Figure 8 and Figure 9). Our research included the rheological characterization of the viscosupplementation product in terms of viscosity, elastic (G′) and viscous moduli (G″), and δ (the ratio of the viscous modulus to the elastic modulus, which indicates the tendency of a fluid to dissipate energy (i.e., more viscous-like) or to store energy (i.e., more elastic-like)). All experiments were performed in conditions designed to simulate the biological environment (37 °C).

The G′ and G″ of the SF samples from the patients treated via supplementation, 5–10 min movement, and PT (2 weeks PT + 4 weeks rest) are presented in Figure 7B,C. G″ > G′ in all analyzed cases.

The viscosupplementation product (Figure 7A) exhibited the rheological behavior of a concentrated HA-CS complex polymeric solution; across the frequency range indicated in the figures below, it demonstrated viscoelastic behavior, with a viscous response at low frequencies (G″ > G′ based on CS and HA’s contribution to the viscous response) and elastic response at high frequencies (G′ > G″). The G′ and G″ of the SF from the patients treated via supplementation, 5–10 min movement, and PT (2 weeks PT + 4 weeks rest) are presented in Figure 5C and Figure 7B. G″ > G′ in all analyzed cases. 

The effect of PT is highlighted in the values of tan δ = G″/G′, a measure of how elastic (tan δ < 1) or plastic (tan δ > 1) the behavior of the tested SF is (Figure 8); tan δ represents the ratio of the viscous to elastic response of a viscoelastic gel (in other words, the energy dissipation potential of the material).

A constant shear stress was applied to the samples in order to evaluate the time needed to adjust the SF (control and supplemented) samples’ responses to the stress conditions (Figure 9). In general, the viscosity of the SF of those with OA was lower than that of the healthy SF samples, which was attributed to the degradation of HA chains and the distribution of low-molecular-weight proteins (e.g., albumin) [56].

Supplementation with an HA-CS complex led to an increase in SF viscosity (Figure 9B), but large variability based on patient specificity was present in our results. Hyaluronan from the supplementation gel could, due to its osmotic contributions and the formation of flow barriers in the limiting layers, serve as an SF viscosity regulator. The time dependency decreased after PT when, in the share rate test condition, the viscosity reached constant values (Figure 9C, P1, P3). Supplementation with no mobilization procedure led to a sharp decline in viscosity in the first few moments after the application of the mechanical loading (Figure 9E). 

### 3.4. Filmogen Characteristics and Bioadhesive Properties

**Figure 10:**
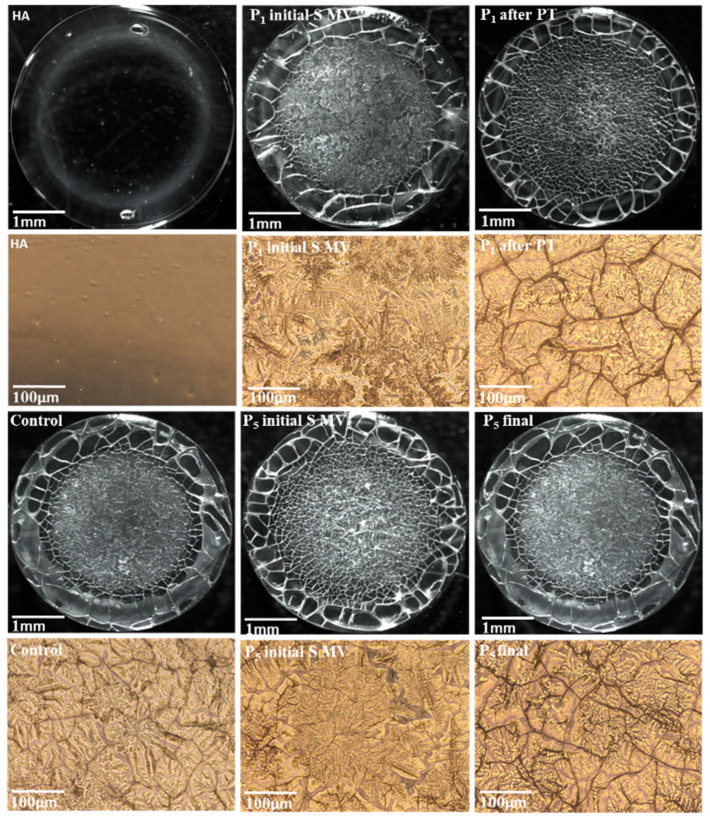
Microscope-derived images of HA and human SF films: control group; Group A (P1 initial S MV and P5 initial S MV); Group B (P1 after PT); Group C (P5 initial S MV); and Group D (P5 final).

The microscope-derived images of dried drops of human SF (taken at low magnification) shown in Figure 10 reveal a heterogeneous deposit. 

The supplementation fluid-based film presented a uniformity, with no cracks or separation. On the contrary, the SF-based films presented two main regions: a glassy film with some separation areas at the drop edge and a fern-shaped crystalline deposit in the central area of the drop [54]. The crystalline area was more pronounced in the SF collected from patients treated via supplementation and 5–10 min movement (P1 and P5), and this result could be linked to the supplementation component’s primary interactions with proteins, glycoproteins, and phospholipids from the SF. The application of PT procedures diminished the clustering tendency, and a uniform center with a diffuse border between the glassy part and the core area (P1 after PT) was observed. 

**Figure 11:**
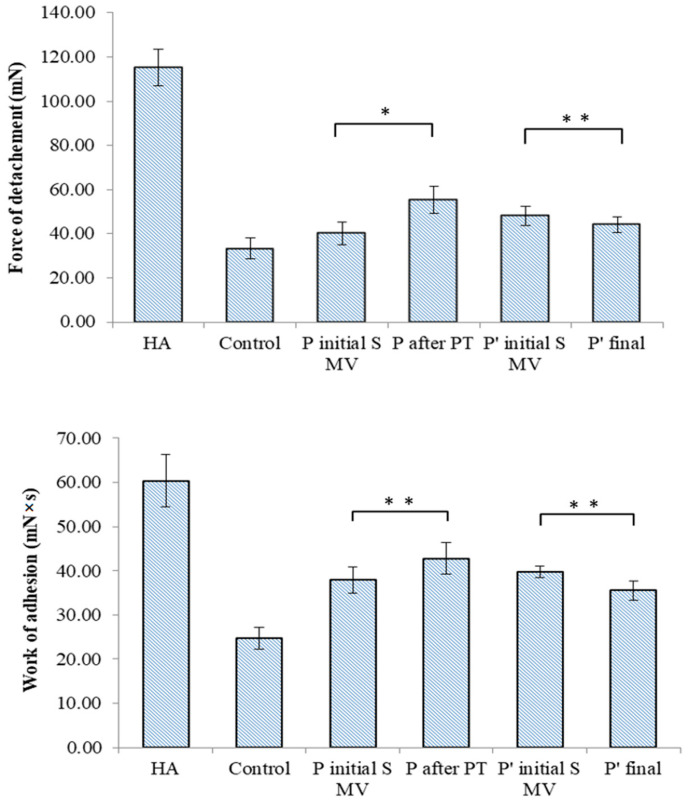
Bioadhesive properties of synovial-fluid-based films tested on a simulating membrane (cellulose membrane) to ascertain the detachment force and the work of adhesion. Values are expressed as the mean of six independent experiments. Each value represents the mean ± standard error of the mean (*n* = 6) (* *p* < 0.05; ** *p* < 0.01).

**Figure 12:**
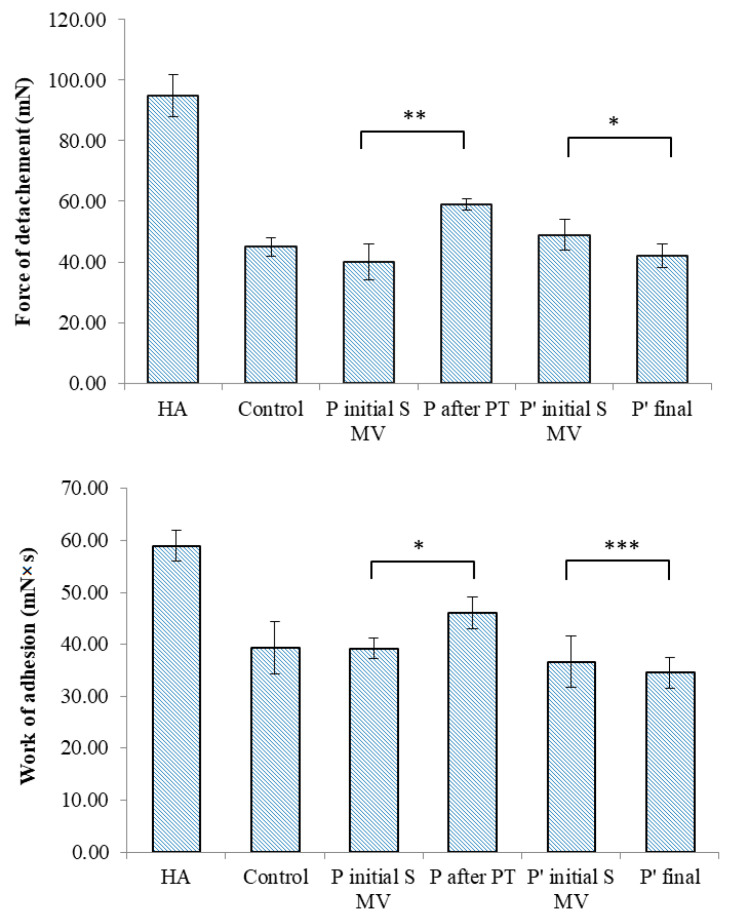
Bioadhesive properties of synovial-fluid-based films tested on chicken cartilage to ascertain detachment force and work of adhesion. Values are expressed as the mean of six independent experiments. Each value represents the mean ± standard error of the mean (*n* = 6) (* *p* < 0.05; ** *p* < 0.01; *** *p* < 0.001).

The TA.XT Plus texture analyzer was used in our in vitro bioadhesion study of the SF-based films, which was carried out using two different simulating membranes: a cellulose membrane (Figure 11) and chicken cartilage (Figure 12). This device provides data related to the maximum force required for the film to detach itself from the simulating membrane, as well as data on the work of adhesion. 

In the tests performed on the cellulose membrane and cartilage, high values of the maximum force of detachment and work of adhesion were recorded for the viscosupplementation product, suggesting strong interactions between the tested SF samples’ components. The component polymers, HA and CS, are bioadhesive and have the ability to spread on the cartilage surface. The SF from the control group presents lower values for the registered bioadhesion parameters, suggesting a variety of interactions between polymeric components less likely to be distributed onto articular cartilage. 

## 4. Discussion

### 4.1. Synovial Fluid Characteristics

SF is a biological gel involved in joint physiology that includes small molecules and macromolecular components generally secreted by cells, like those of HA (a linear, negatively charged polysaccharide), lubricin, proteins, and surface-active phospholipids (especially phosphatidylcholine) [57]; these components are concentrated in the joint cavity, and changes in the lubrication or structures of joints lead to damage to articulating cartilage surfaces [58]. In the human body, HA has been identified as a sodium slat (HAs) fenced by water molecules, forming both highly hydrophilic molecules and a sphere. The architecture of this natural biopolymer is responsible for its excellent physicochemical and biological properties, such as its high water-binding capacity and viscoelastic behavior [59], non-immunogenic and non-inflammatory characteristics, and totally biodegradable features [60]. HA has been intensively studied for various medical applications, such as wound healing, drug delivery, synovial fluid substitution, injection implants, etc.

Other components in SF include proteins (total protein content is about 2%), proteoglycan 4 (PRG4–lubricin), and surface-active phospholipids (SAPLs), especially phosphatidylcholine [61]. The proteins present in SF include albumin, serotransferrin, apolipoprotein A, and several immunoglobulins [62]. Secreted lubricin, a glycoprotein on cartilage surfaces, is also present in synovial joints, and this component has a role in lubrication and the prevention of protein and cell adhesion [63,64]. Changes in SF composition and characteristics give indications about the status or pathological condition of the joint. 

Regarding the pH of SF, as mentioned in the Results section, the values measured in our study are comparable with those presented in other articles [51]. 

Some authors attribute synovial fluid acidification in OA to initial joint inflammation and the activation of cathepsin K, an acidic cysteine endoproteinase that degrades collagen in cartilage, also influencing the synovial fluid. The activation of cathepsin K, overexpressed in OA joints, is facilitated by phenotypically altered chondrocytes and stresses the intimate relationship between the synovial fluid microenvironment and cartilage, contributing to the progressive degeneration of the cartilage [65].

High concentrations of glucose have been associated with OA. Under limited amounts of oxygen, glucose is converted to lactate or pyruvate, molecules involved in the Krebs cycle. Anaerobic glycolysis is stimulated in OA-affected chondrocytes, and glucose is released in synovial fluid [66]. Generally, in our study, supplementation and PT led to reductions in the glucose concentration.

### 4.2. Fourier-Transform Infrared (FTIR) Data 

Various methods have been used in order to characterize healthy or pathological SF, from biochemical analysis and structural evaluations of the macro- and micromolecular components to mechanical tests. Fourier-transform infrared (FTIR) spectroscopy is performed to differentiate between healthy SF and SF from OA-affected joints, and this method has been considered as a screening approach for diagnosis. 

In [67], chondroitin sulfate (CS) profiles in SF from a patient with a representative knee injury and aggrecan purified from the same SF sample were compared, and modifications in sulfated glycosaminoglycan were identified. Regarding FTIR spectra, differences in the C=O stretching vibrations (amide I region, 1584–1720 cm^−1^) and C–O stretching vibrations of carbohydrate moieties (HA, 984–1140 cm^−1^) have been identified, indicating that FTIR spectroscopy is a suitable alternative analytical method for detecting OA-related changes in SF compared to the current immunoassay and chromatographic methods, which often require more sophisticated instrumentation and sample processing [68]. 

ATR-FTIR data have revealed the important effects PT has on the interactions between the viscoelastic product and SF: the supplementation gel, rich in HA and CS, can interact with synovial fluids to form a homogenous system without any separation and clustering, working as a single-phase synovial-like fluid [55]. 

As a final remark, IR spectroscopy could be used as a primary screening method for evaluating the treatment course in cases involving patients with moderate KOA.

### 4.3. Rheological Properties

The rheology of SF influences the tribology of both healthy and pathological joints. Based on the organization of proteins and HA and their interactions, the functioning of joints is strongly related to the condition of the SF. The rheological behavior of SF is changed in arthritis or OA, presenting decreased viscoelasticity [69]. It has been found that the intra-articular injection of HA restores SF’s viscoelasticity in patients with KOA, though there are some biological consequences that come with this form of treatment: decreases in pro-inflammatory cytokines and lining cells, lymphocytes, and macrophages, as well as an increase in the number of fibroblasts and the amount of collagen after supplementation with HA gels, were observed [70,71].

In the present study, the elastic and viscous moduli values varied from one patient to another (control group, Figure 7A), which points toward the fact that, in OA, many factors contribute to the variability of the rheological properties of SF, including the modification of inflammatory components, joint geometry, and pathology history. Joint alteration affects the properties of the SF to a larger extent; in this study, the values ranged from 0.1 Pa to 10 Pa, ten times less than the supplementation fluids based on HA and CS.

Figure 7B,C show that the elastic modulus values increased, even doubling in some cases, indicating a uniform distribution of the HA hydrogel among the components of SF, a process stimulated by the movement conditions. The SF’s lower elastic behavior at the physiological shear rates is evidence of the hyaluronate and CS’s interaction with proteins, especially lubricin; lubricin decreases the degree of entropy of an otherwise stiffer molecule. PT treatment contributed to a homogenous distribution and new organization of the proteins and polysaccharides in the SF, and G′ and G″ slightly decreased (Figure 7C), with an increased contribution of the viscous modulus being noted. The presence of lubricin and other glycoproteins helps synovial fluid to store and dissipate the energy generated upon impact with a normal walking gait and assures adequate lubrication together with other HA-binding proteins. For those who were not recommended to partake in PT (Figure 7D,E), the G′ and G″ profiles were non-uniform in our frequency sweep tests, suggesting a high sensitivity to the measurement conditions [72].

The values for tan δ were generally higher than 1 for all of the tested SF samples, indicating a more plastic fluid. When applying a load to the synovial gel, some part of the applied load is dissipated by energy dissipation mechanisms (such as segmental motions) in the bulk of the synovial polymeric network, and the other part of the load is stored in the polymeric component derived from the SF. The energy is released upon the removal of the load. A decrease in the tan δ value indicates a more elastic biological gel, and when applying a load, it has more potential to store the load rather than dissipating it; this effect is more pronounced in SF after PT (Figure 8C). On the other hand, an increase in the tan δ value is associated with greater energy dissipation potential in supplemented SF and can be produced with PT, with such behavior being attributed to the presence of intense inflammatory processes that change the synovial proteins’ interactions with HA.

SF presents a time-dependent viscosity, as is the case with thixotropic fluid, and this behavior is emphasized in OA [73]. In designing a combination of treatments that involve lubricants, the viscosity of the viscosupplementation fluid should be high enough to provide a continuous fluidic interface in the contact area but not too large to create friction due to viscous shear. The knee has a role in carrying out low-speed and high-load actions, and greater viscosity is desirable [74]. Furthermore, the synovial membrane that separates SF from plasma allows small globular proteins (albumin and globulins) to freely interchange between blood and SF, and this process is more intense in rheumatoid arthritis. For such patients, a higher albumin concentration in their SF and a smaller than normal albumin concentration in their plasma can be found simultaneously [75]. 

### 4.4. Filmogen Characteristics and Bioadhesive Properties

Through analyzing the filmogen characteristics of human SF (Figure 10), we observed the integral distribution of the supplementation fluid among macromolecules in SF. HA chains have associative abilities and create a structural matrix for SF’s lipidic components. An increased content of HA and CS chains leads to the absorption of phospholipid vesicles and their organization into brush-like structures that facilitate the lubrication mechanisms in the joint [76]. Proteins and glycoproteins are intercalated in the formed matrix. Therefore, viscosupplementation increases the concentration of anchoring sites and consolidates the architecture of the entire SF. Matrix structuration, along with some inhomogeneities, was observed in the SF from the viscosupplemented group who partook in 5–10 min movement and had 6 weeks of rest. 

Bioadhesion assays express the composition of SF and the interactions within SF, and they are especially useful for gaining insights into polymeric networks and their changes in pathological conditions, the composition of the administrated supplementation gels, and additional procedures that contribute to the interfusion and structuring of proteins, polysaccharides, glycoproteins, and phospholipids. The bioadhesive nature of proteins and polysaccharides is attributed to their hydrophilic networks, which have numerous functional polar groups (e.g., –COOH, –OH, –NH_2_, and –SO_4_), and their chain lengths, as well as the biopolymers’ degree of hydration and concentration [77,78]. The adhesion mechanism is also dependent on ionic interactions, hydrogen bonds, and van der Waals forces [79,80].

In this study, it was observed that the supplementation improved the bioadhesive properties of the SF samples and that PT was favorable for the distribution of macromolecules, their association with proteins and glycoproteins, and the anchoring of phospholipids, potentially increasing the lubrication characteristics. During movement, in OA-affected joints, the SF enters and transforms the gaps in the cartilage, and the pressure increases as the space converges, creating a hydrodynamic lift and forcing the surfaces apart like a wedge. The thickness of the film formed by the fluid should be slightly greater than the surface roughness in order to minimize asperity contact. The viscous product works in synergy with lubricin, while PT contributes to the supplementation fluid distribution and structuring and finally improves the SF’s ability to spread onto a textured cartilage surface. 

This study had some limitations since only one single-dose HA product was used for all patients, regardless of age, gender, and weight in Groups 2 and 3. The clinical PT protocol was tested before this study and applied to the patients in Group 2 (P1, P2, and P3). Another limitation of this study was the use of a single 10-day PT protocol, and future studies should focus on treatment protocols with longer periods in different combinations with new technologies such as TECAR radiofrequency, deep oscillation, etc. Another important limitation was the small number of patients evaluated and the evaluation interval, which was only 6 weeks. Because changes in the physicochemical properties of the synovial fluid of patients viscosupplemented with HA and treated with PT were observed, future studies will be performed with a larger number of patients and with multiple clinical and rheological evaluation intervals to unlock the potential of this study.

## 5. Conclusions

KOA is a progressive and disabling condition that requires new treatment strategies due to the fact that the existing ones have yielded questionable results, especially in terms of symptom control. This study summarizes an investigation of synovial fluid samples collected from patients with moderate KOA treated using a combined therapy based on supplementation gel (based on either hyaluronic acid or chondroitin sulfate) and PT. Several changes in SF characteristics (pH normalization and glucose concentration reduction) were identified as a result of the supplementation and PT’s combined effect on the composition and organization of the synovial fluid. The viscosupplementation product exhibits the rheological behavior of an HA-CS complex polymeric solution, with a viscous response at low frequencies and elastic response at high frequencies. PT treatment contributes to a homogenous structuring of the HA-CS complex in synovial fluid, its interaction with proteins and glycoproteins, and the anchoring of lipidic components, also exerting some effects on the viscous modulus and viscoelastic rheological behavior of SF. The SF samples taken from the knees of patients with moderate OA were thixotropic fluids with a time-dependent viscosity; the dependency slightly decreased under the effect of PT, and the viscosity became constant. The viscosupplementation improved the bioadhesive properties of the SF, indicating that, along with PT, viscosupplementation is favorable for protein and phospholipid anchoring and uniform film forming, with potential impacts on lubrication. PT can assist the non-surgical treatment of KOA based on its effects on the physical–chemical characteristics of SF, biorheology and bioadhesion, and glycoprotein distribution on the cartilage surface, leading it to have a positive effect on patients. Having an understanding of the predisposing factors of KOA, the occurrence of acute inflammatory phenomena, and the perpetuation of the mechanisms that latently maintain chronic inflammation that develops over time and has a destructive effect on articular cartilage can help in limiting the negative effects of KOA, delaying its evolution, and optimally combating the phenomena that maintain the vicious circle: inflammation → enzyme production → chondrolysis → inflammation.

## Figures and Tables

**Table 1:** Characteristics of the tested SF samples.

No.	Encoded	Characteristics	Solid (mg/mL)	pH	Color	Clarity	Glucose (mg/dL)
1.	HA	Viscosupplementation material	76	7.22	Clear	Transparent	0
2.	Control 1	Patient with no procedure	0.88	7.14	Amber–Yellow	Transparent	144 ± 4
3.	Control 2	Patient with no procedure	0.86	7.18	Amber–Yellow	Transparent	143 ± 1
4.	Control 3	Patient with no procedure	0.83	6.89	Yellow	Transparent	101 ± 2
5.	P1 Initial S MV	Patient 1 initial (supplemented) 5–10 min movement	0.40	7.03	Yellow	Transparent	110 ± 3
6.	P2 Initial S MV	Patient 2 initial (supplemented) 5–10 min movement	0.68	6.88	Amber–Yellow	Transparent	61 ± 2
7.	P3 Initial S MV	Patient 3 initial (supplemented) 5–10 min movement	0.88	7.14	Amber–Yellow	Transparent	53 ± 1
8.	P1 Final	Patient 1 after PT (2 weeks PT + 4 weeks rest)	0.66	7.32	Amber–Yellow	Transparent	99 ± 5
9.	P2 Final	Patient 2 after PT (2 weeks PT + 4 weeks rest)	0.68	7.28	Amber–Yellow	Transparent	67 ± 1
10.	P3 Final	Patient 3 after PT (2 weeks PT + 4 weeks rest)	0.64	7.42	Amber–Yellow	Transparent	69 ± 2
11.	P4 Initial S MV	Patient 4 initial (supplemented) 5–10 min movement	0.72	7.19	Amber–Yellow	Transparent	104 ± 2
12.	P5 Initial S MV	Patient 5 initial (supplemented) 5–10 min movement	0.76	6.87	Amber–Yellow	Transparent	120 ± 4
13.	P6 Initial S MV	Patient 6 initial (supplemented) 5–10 min movement	0.76	7.24	Amber–Yellow	Transparent	59 ± 2
14.	P 4 final	Patient 4 final (6 week rest)	0.74	7.32	Amber–Yellow	Transparent	113 ± 3
15.	P 5 final	Patient 5 final (6 week rest)	0.62	7.51	Amber–Yellow	Transparent	128 ± 3
16.	P 6 final	Patient 6 final (6 week rest)	0.70	7.37	Amber–Yellow	Transparent	70 ± 2

**Table 2:** Typical FTIR bands for HA, CS, and Kombihylan^®^.

Sample	Typical Bands (cm^−1^)	Functional Groups	Ref.
HA	3296 cm^−1^	stretching vibration of the hydrogen bond from –NH– and of –OH intra-/inter-molecular	[52,53]
	2855 cm^−1^	–CH_2_ symmetric stretching vibration	
	1596 and 1405 cm^−1^	COO^−^ symmetric and asymmetric vibration	
	1023 cm^−1^	C–O–C hemiacetalic system saccharide units	
CS	>3000 cm^−1^	OH stretching vibration	[54]
	1372.58 cm^−1^	sulfate	
	1227.81 cm^−1^	S=O group	
	850 cm^−1^	C–O–S vibration	
Kombihylan^®^	both polymers’ groups with highlighted peaks attributed to HA		

## Data Availability

Data are contained within the main text of the article.
